# Protocol for integrative analysis of transcription factor-nucleosome interactions using SeEN-seq and cryo-EM structure determination

**DOI:** 10.1016/j.xpro.2025.104295

**Published:** 2025-12-26

**Authors:** Wataru Kobayashi, Alicia K. Michael, Siwat Ruangroengkulrith, Maximilian Kümmecke, Kikuë Tachibana

**Affiliations:** 1 Department of Totipotency, Max Planck Institute of Biochemistry (MPIB), Munich, Germany; 2 Institute of Science and Technology Austria (ISTA), Klosterneuburg, Austria

**Keywords:** High-throughput screening, Microscopy, Structural biology

## Abstract

Pioneer transcription factors (TFs) possess the ability to read out DNA motifs embedded within nucleosomes, driving changes in gene expression during cellular differentiation and reprogramming. Here, we present selected engagement on nucleosome sequencing (SeEN-seq), a protocol designed to systematically identify potential TF-binding sites on the nucleosome. We describe steps for nucleosome library assembly, SeEN-seq assay, and cryoelectron microscopy (cryo-EM) sample preparation. This protocol facilitates the preparation of homogeneous pioneer TF-nucleosome complexes for cryo-EM structure determination using single-particle analysis.

For complete details on the use and execution of this protocol, please refer to Michael et al.^1^

## Highlights

• Instructions for the preparation of an SeEN-seq DNA library by EcoRV digestion
• Steps for purification using either the anion exchange column or Prepcell apparatus
• Procedures for SeEN-seq data analysis
• Guidance of TF-nucleosome complex preparation for cryo-EM analysis

## Abstract (editor highlights)

Publisher’s note: Undertaking any experimental protocol requires adherence to local institutional guidelines for laboratory safety and ethics.

## Graphical abstract

**Figure:**
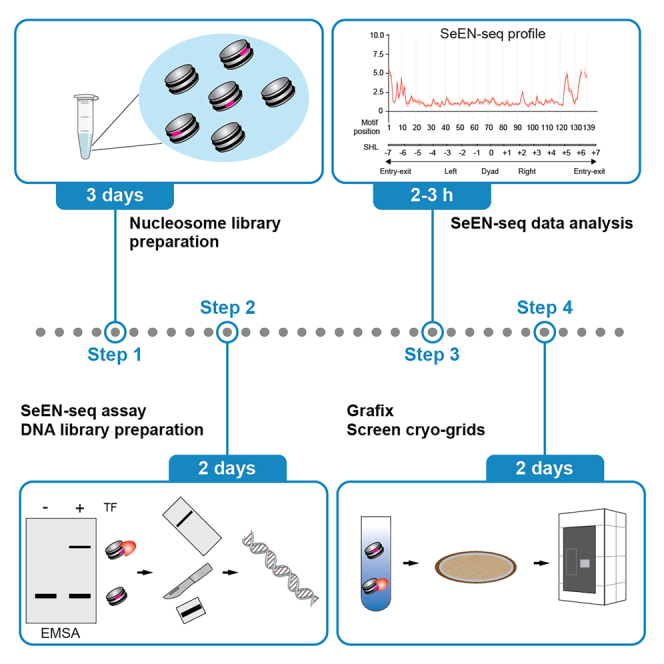

## Before you begin

**Figure 1:**
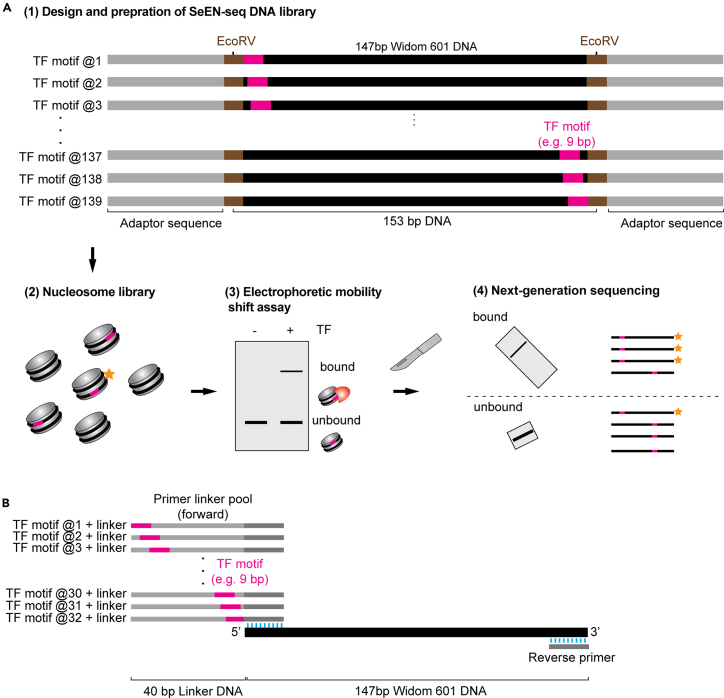
Workflow of SeEN-seq (A) A nucleosome library is reconstituted using Widom 601 DNA containing the TF motif of interest, tiled with 1 bp intervals. Nucleosomes selectively targeted by the TF are separated by PAGE and identified by sequencing. The DNA sequences targeted by the TF are specifically enriched in the bound fraction compared to the unbound fraction. The ratio of TF-bound to unbound reads of each DNA construct is then calculated to identify potential TF binding sites. Stars indicate a representative DNA sequence preferentially targeted by the TF. (B) Schematic illustrating the amplification of a SeEN-seq library including linker DNA. TF motif is indicated in pink, tiled through a linker sequence, typically an extension of the Widom 601 sequence.^2^ Length of the linker is limited by primer synthesis, where primers containing the TF motif tiled at 1bp intervals are synthesized, with an ∼20–30bp overlapping region (blue lines) with the Widom 601 sequence. The library is then amplified by PCR with the TF-linker primers mixed at equimolar concentrations using the Widom 601 as a template. To generate the fully tiled library, the ‘linker’ library is combined with the synthesized W601 library at equimolar concentrations for each construct.

Selected Engagement on Nucleosome Sequencing (SeEN-seq)^1^ is a quantitative *in vitro* assay that systematically maps all possible transcription factor (TF)-binding positions on a nucleosome at single-base pair resolution. SeEN-seq comprises tiling the TF motif at 1 base pair (bp) intervals across 147 bp of the Widom 601 (W601) nucleosome positioning sequence,^2^ reconstituting a nucleosome library, and separating TF-bound and unbound nucleosomes using electron mobility shift assay (EMSA) followed by sequencing (Figure 1A). Using this protocol, we mapped several TFs, including OCT4,^1^ SOX2,^1^ OCT4-SOX2,^1^ CLOCK-BMAL1,^3^ MYC-MAX,^3^ and NR5A2.^4^ The resulting data enabled us to prepare homogenous samples to determine the cryogenic electron microscopy (cryo-EM) structure of TF-nucleosome complexes.^1^^,^^3^^,^^4^

### Innovation

The nucleosome, the basic unit of chromatin, is a protein-DNA complex in which ∼147 bp of DNA are wrapped around a histone octamer. The histone–DNA interaction restricts DNA accessibility, but TFs need to access their target motifs in the context of chromatin to regulate gene expression. Notably, a subclass of TFs known as pioneer TFs is capable of binding to the closed chromatin. However, the rotational setting of TF motifs within the nucleosomal DNA critically impacts TF binding. To understand the molecular mechanisms by which TFs engage chromatin, it is essential to systematically characterize the binding mode of distinct TF families on the nucleosome.

In a previous study, NCAP-SELEX^5^ enabled high-throughput profiling of TF binding preferences using nucleosomes reconstituted with randomized DNA sequences. This approach revealed the general positional preference of TF binding on the nucleosome. SeEN-seq is an alternative systematic approach that offers a base-pair resolution map of TF-nucleosome interactions by tiling defined motifs across the 147 bp W601 DNA and directly separating bound and unbound nucleosomes in a single-step assay. SeEN-seq also accommodates composite motifs, allowing investigation of TF cooperativity. Importantly, the identified DNA sequences in SeEN-seq are suitable for cryo-EM analysis, enabling high-resolution structural analysis. Together, SeEN-seq offers a mechanistically and structurally tractable *in vitro* assay to investigate TF binding preferences and chromatin engagement with high resolution.

### Designing a DNA library for SeEN-seq

**Timing: 1–2 h**

This section describes the design a SeEN-seq DNA library.

1. Delete the 147 bp Widom 601 (W601) nucleosome positioning sequence locally and replace an equal length of your TF motif of interest, shifting the motif by 1 bp across the entire W601 sequence.

***Note:*** The number of sequences in the SeEN-seq DNA library will depend on the length of the inserted TF motif. Each designed 147 bp W601 DNA sequence containing the TF motif is flanked by EcoRV restriction sites (6 bp each), resulting in 153 bp of DNA upon EcoRV digestion (Figure 1A). These DNA fragments can be ordered from any gene synthesis provider. We typically order 300 bp of gene fragments with adaptors from Twist Bioscience. Any randomized DNA sequence can be used as the adaptor sequence. If ordering gene fragments from Twist Bioscience, request the maximum amount per construct (typically 700–1000 ng). Please see Table S1 for the example of SeEN-seq DNA library.

**CRITICAL:** In some cases, replacing W601 with the TF motif generates an EcoRV restriction site, resulting in a shorter DNA fragment that cannot be incorporated into the nucleosome library. If this occurs, either exclude those constructs or use a different blunt-end restriction enzyme site for DNA digestion.

2. To make your work easy, you can generate a SeEN-seq DNA library using our script. The script can be found in make_SeENseq_template.r of the GitHub repository.
3. To probe TF binding within the linker DNA, you can extend the core Widom 147 bp sequence by 20–30 bp of linker sequence and directly order a synthesized library. Alternatively, it is possible to add these additional ‘linker’ motifs using primers that extend the 5′ flanking DNA by 40 bp, with the TF motif tiled at 1 bp intervals within the linker, using the Widom 601 (without motif) as a PCR template (Figure 1B). This smaller linker library can then be mixed at equimolar concentration with ‘internal’ purified motifs that require gene synthesis. The examples for these primers are as follows:

40 bp Linker sequence: tatccgactggcaccggcaaggtcgctgttcaatacatgc

Example primer: *tatccgact*ggcaccggcaaggtcgctgttcaatacatgc**acaggatgtatatatctgacacgtgcct***Italic* = replace with TF motif

**bold** = overlap with W601.

### Amplification of 153 bp W601 carrier DNA by polymerase chain reaction

**Timing: 6–8 h**

Because motif-containing sequences are limited in quantity, standard W601 DNA is therefore used to increase nucleosome assembly yield. Purification of the 153 bp W601 DNA containing the TF motif of interest (introduced in the previous chapter) will be detailed later in the “step-by-step method details.”

4. Purify the pGEM plasmid DNA containing the 153 bp W601 sequence (single repeat) using a Midiprep or Maxiprep kit (Qiagen).
5. To amplify the 153 bp W601 carrier DNA, prepare 9.6 mL of PCR reaction for two 96-well plates. The PCR reaction components are as follows:

**Table:** 

Reagent	Volume (uL)	Stock	Final
pGEM-153bp W601 (single repeat)	192	100 ng/ul	2 ng/ul
Phusion Polymerase	96	-	-
Forward primer	48	200 uM	1 uM
Reverse primer	48	200 uM	1 uM
Phusion HF buffer	1920	5x	1x
dNTPs	76.8	25 mM	0.2 mM
DMSO	192	100%	2%
ddH_2_O	7027.2	-	-
**Total**	9600

***Note:*** Mix everything in a 15 mL tube on ice during these preparations.

6. Dispense 50 μL of the PCR reaction mixture into each well and seal the plate. Use a multichannel pipette or multipipette dispenser if available to speed up the process.
7. Run PCR using the following cycling conditions:

**Table:** 

Steps	Temperature	Time	Cycles
Initial Denaturation	94°C	3 min	1
Denaturation	94°C	15 sec	35–50 cycles
Annealing	62°C	30 sec	
Extension	72°C	30 sec	
Final extension	72°C	5 min	1
Hold	4°C	forever

8. Spin down the plates.
9. Pool all PCR reactions into a 50 mL tube and keep the sample on ice.
10. Check the yields and purity of PCR product by loading samples on a 2.5% agarose gel containing SYBR Safe (1:10,000 dilution):
  a. 100 bp DNA ladder.
  b. 10 μL of PCR products mixed with 2 μL of 6x loading dye.

### Purification of 153 bp W601 carrier DNA

**Timing: 4–5 h**

Purify the amplified W601 DNA using either an anion exchange column or the Model 491 Prepcell apparatus (BIO-RAD). We normally obtain 200–300 μg after purification.

***Note:*** For DNA purification with an anion exchange (Q) column, see Step 11. For DNA purification using the Model 491 Prepcell apparatus, see Step 12.

11. DNA purification with an anion exchange (Q) column
  a. Directly after PCR amplification in 96-well plates and confirmation of the correct amplicon, combine all 50 μL reactions into a clean multichannel pipette reservoir.
  b. The PCR reactions will be directly injected onto an FPLC purification system to perform anion exchange. Using a syringe, aspirate the entire volume and filter using a 0.2 μM or 0.45 μM filter into a 50 mL conical tube.
  c. Load sample directly to an ion exchange column (HiTrap Q, 5mL) with the following program:
    i. HiTrap Q column Buffer A: 20 mM Tris-HCl (pH 7.5), Buffer B: 20 mM Tris-HCl (pH 7.5) + 1 M NaCl
    ii. Wash 15 column volumes (CV)
    iii. Gradient elution, 10 CV, 0%–100% Buffer B
  d. The desired DNA peak of ∼150 bp will elute at ∼57 mS/cm or 80% B.
  e. Run 2.5 % agarose gel (or non-denaturing PAGE, see Step 12t) to assess DNA purity.
  f. Combine fractions and concentrate with spin-filter device (10–30K MWCO). To remove high NaCl and exchange the buffer, dilute the sample with 10 times volume of HiTrap Q column Buffer A and reconcentrate. Repeat this process at least three times.
  g. Concentrate to a final volume ∼100–200 μL and measure DNA concentration by absorbance at 260 nM.

***Note:*** A final concentration > 1.0 mg/ml is recommended.

**Pause point:** Store purified DNA at −20°C until nucleosome reconstitution.

12. DNA purification using the Model 491 Prepcell apparatus
  a. After confirming successful DNA amplification, add 400 μL of TE buffer to a 50 mL tube, bringing the total volume to 10 mL.
  b. Add 10 mL of Phenol: Chloroform: Isoamyl Alcohol (25:24:1, v/v, pH 8.0) and mix by vortexing for 3–5 seconds.
    ***Note:*** Handle phenol and chloroform in a chemical fume hood due to their toxicity.
  c. Centrifuge at 3,500 rpm (2,451 x g) for 20 min at 20°C.
  d. Carefully transfer the aqueous (upper) phase into a Beckman centrifuge tube (50 mL Polypropylene Bottle).
  e. Add 1 mL of sodium acetate (3M, pH 5.2) and 25 mL of 100% ethanol. Mix gently.
  f. Incubate at −80°C for 30 min or at −20°C overnight (>16 hours).
  g. Centrifuge at 15,000 rpm (27,216 x g), for 30 min at 4°C using a JA-25.50 rotor in a Beckman centrifuge (Avanti).
  h. Discard the supernatant.
  i. Add 5 mL of 70% ethanol.
  j. Centrifuge at 15,000 rpm (27,216 x g) for 15 min at 4°C.
  k. Discard the supernatant.
  l. Remove any residual ethanol with a pipette.
  m. Air-dry the pellet with the tube open for 15–30 min.
    ***Note:*** Complete drying is not necessary; avoid over-drying the pellet.
  n. Resuspend the DNA pellet in 300 μL of 1xTE buffer.
  o. Prepare a 6% non-denaturing polyacrylamide gel in 0.2xTBE within the PrepCell gel-tube assembly. A gel height of approximately 6.5 cm. Use 0.2xTBE and TCS buffer EDTA (+) for running buffer and sample elution buffer, respectively. Set up Model 491 Prep Cell (BIO-RAD) according to the manufacturer’s instructions (https://www.bio-rad.com/webroot/web/pdf/lsr/literature/M1702925C.PDF).
  p. Set the PowerPac Universal Power Supply (BIO-RAD) to 10 W and pre-run the gel for 1 h at a flow rate of 1.0 mL/min in the cold room.
  q. Load the DNA sample mixed with 5% sucrose into the Prepcell and start the run at 10 W (defined as time point 0 min).
  r. Collect the samples with 9 mL/fractions at a flow rate of 1.0 mL/min from 0 to 40 min
  s. Collect the samples with 2.25 mL/fraction at a flow rate of 1.5 mL/min from 40 to 90 min
    ***Note:*** W601 DNA will be eluted around 60 min.
  t. Analyze collected fractions by a 10% non-denaturing PAGE (0.2xTBE), running at 150V for 1 h (Mini-PROTEAN Tetra Cell, BIO-RAD).
  u. Pool the fractions containing the W601 DNA and concentrate them using an ultra-centrifugal filter (30 kDa cutoff).
  v. Measure DNA concentration by absorbance at 260 nm.
    ***Note:*** As above, a final concentration > 1.0 mg/ml is recommended.
    **Pause point:** Store purified DNA at −20°C until nucleosome reconstitution.

## Key resources table

**Table:** 

REAGENT or RESOURCE	SOURCE	IDENTIFIER
**Chemicals, peptides, and recombinant proteins**		

Tris	Sigma-Aldrich	252859
HEPES	Sigma-Aldrich	H6147
EDTA	Sigma-Aldrich	03677
Sodium chloride	Sigma-Aldrich	S5886
Potassium chloride	Merck	P9541-1KG
Zinc chloride	Sigma-Aldrich	208086
Magnesium chloride	Sigma-Aldrich	208337
sodium acetate	Sigma-Aldrich	S5636
β-Mercaptoethanol	Sigma-Aldrich	M3148
DTT	Roche	10708984001
TCEP	Sigma-Aldrich	C4706
Guanidine hydrochloride	Sigma-Aldrich	G3272
Agarose	Thermo Fisher Scientific	16520050
Phenol:chloroform:isoamyl alcohol 25:24:1	Invitrogen	15593031
SYBR Safe	Thermo Fisher Scientific	A45204
SYBR Gold	Invitrogen	S11494
TEMED	Sigma-Aldrich	T9281-25ML
40% acrylamide and bis-acrylamide solution, 29:1	Bio-Rad	1610146
APS	Sigma-Aldrich	A3678
EcoRV-HF	New England Biolabs	R3195M
Sucrose	Sigma-Merck	S9378
BSA (Bovine serum albumin)	Roche	10715859103
pGEM-T easy vector system	Promega	A1360
Paraformaldehyde 32% Aqueous Solution EM Grade	Electron Microscopy Sciences	15714
Glutaraldehyde	Carl Roth	4157.1

**Critical commercial assays**		

QIAquick Gel Extraction kit	QIAGEN	28704
QIAquick PCR Purification Kit	QIAGEN	28106
QIAGEN Plasmid Kits for Plasmid DNA Extraction	QIAGEN	12143, 12162
Phusion High-Fidelity DNA Polymerase	New England Biolabs	M0530L
Amicon Ultra Centrifugal Filters, 30 kDa MWCO	Millipore	UFC803024
PD-10 desalting column	Cytiva	17085101
DNA LoBind tube	Eppendorf	0030108051
NEBNext Ultra II DNA Library Prep Kit	New England Biolabs	E7645S
Quantfoil 1.2/1.3 holy carbon copper grids	Electron Microscopy Sciences	Q250-CR1.3

**Oligonucleotides**		

Forward primer: 5′-ATCCTGGAGAATCCCGGTG	This paper	N/A
Reverse primer: 5′-ATCACAGGATGTATATATCTGACACGTG	This paper	N/A

**Recombinant DNA**		

pGEM-153 bp W601 (single repeat)	Gassler et al.^6^	N/A

**Recombinant proteins**		

Mouse ESRRB	Gassler et al.^6^	N/A
Mouse histones	Gassler et al.^6^	N/A

**Software and algorithms**		

R: A language and environment for statistical computing	R Core Team^7^	https://www.R-project.org/
Tidyverse	Wickham et al.^8^	https://www.tidyverse.org/
GenomicRanges	Lawrence et al,^9^	https://bioconductor.org/packages/release/bioc/html/GenomicRanges.html
QuasR	Gaidatzis et al.^10^	https://www.bioconductor.org/packages/release/bioc/html/QuasR.html
SummarizedExperiment	Morgan et al.^11^	https://bioconductor.org/packages/release/bioc/html/SummarizedExperiment.html
Writexl	Ooms^8^	https://cran.r-project.org/web/packages/writexl/index.html

**Other**		

ÄKTA chromatography system	Cytiva	N/A
HiTrap Q HP, 5 mL column	Cytiva	17115301
MonoQ 5/50 GL	Cytiva	17-5166-01
NanoDrop	Thermo Fisher Scientific	N/A
Model 491 Prep Cell	Bio-Rad	1702928
PowerPac Universal Power Supply	Bio-Rad	1645070
Next-generation sequencer	N/A	N/A
Gradient Master	BIOCOMP	N/A
Ultra-centrifugation	Beckman	N/A
SW-41Ti rotor	Beckman	N/A
Qubit	Thermo Fisher Scientific	N/A
Tapestation	Agilent	N/A
Vitrobot	Thermo Fisher Scientific	N/A
200kV cryo-transmission electron microscope (e.g., Talos Arctica, Glacios)	N/A	N/A

## Materials and equipment

**Table:** TE buffer

Reagent	Final concentration	Amount
1 M Tris-HCl, pH 8.0	10 mM	5 mL
0.5 M EDTA, pH 8.0	0.1 mM	0.1 mL
ddH_2_O	N/A	Add to 500 mL
**Total**	**N/A**	**500 mL**

Autoclave, then store at room temperature (20°C–25°C) up to 3 months.

**Table:** TCS EDTA (+) buffer

Reagent	Final concentration	Amount
1 M Tris-HCl, pH 7.5	20 mM	10 mL
0.5 M EDTA, pH 8.0	1 mM	1 mL
DTT	1 mM	77 mg
ddH_2_O	N/A	Add to 500 mL
**Total**	**N/A**	**500 mL**

Filter the buffer using a 0.22 μm filter unit. Store at 4°C. This buffer should be freshly prepared.

**Table:** TCS EDTA (−) buffer

Reagent	Final concentration	Amount
1 M Tris-HCl, pH 7.5	20 mM	10 mL
DTT	1 mM	77 mg
ddH_2_O	N/A	Add to 500 mL
**Total**	**N/A**	**500 mL**

Filter the buffer using a 0.22 μm filter unit. Store at 4°C. This buffer should be freshly prepared.

**Table:** 10 x EMSA buffer BSA (+)

Reagent	Final concentration	Amount
1 M Tris-HCl (pH7.5)	200 mM	20 μL
100 mM MgCl_2_	10 mM	10 μL
100 μM ZnCl_2_	10 μM	10 μL
100 mM DTT	10 mM	10 μL
20 mg/ml BSA	1 mg/ml	5 μL
ddH_2_O	N/A	45 μL
**Total**	**N/A**	**100 μL**

Store at 4°C. This buffer should be freshly prepared.

**Table:** 10 x EMSA buffer BSA (−)

Reagent	Final concentration	Amount
1 M Tris-HCl (pH7.5)	200 mM	20 μL
100 mM MgCl_2_	10 mM	10 μL
100 μM ZnCl_2_	10 μM	10 μL
100 mM DTT	10 mM	10 μL
ddH_2_O	N/A	50 μL
**Total**	**N/A**	**100 μL**

Store at 4°C. This buffer should be freshly prepared.

**Table:** Gel extraction buffer

Reagent	Final concentration	Amount
5 M Ammonium acetate	500 mM	5 mL
1 M magnesium acetate	10 mM	0.5 mL
500 mM EDTA (pH 8.0)	1 mM	0.1 mL
10% SDS	0.1%	0.5 mL
ddH_2_O	N/A	Add to 50 mL
**Total**	**N/A**	**50 mL**

Store at room temperature (20°C–25°C) up to 3 months.

**Table:** PD-10 elution buffer

Reagent	Final concentration	Amount
1 M HEPES-NaOH (pH 7.5)	10 mM	1 mL
100 mM DTT	1 mM	1 mL
1 mM ZnCl_2_	1 μM	0.1 mL
ddH_2_O	N/A	Add to 100 mL
**Total**	**N/A**	**100 mL**

Store at 4°C. This buffer should be freshly prepared.

**Table:** Grafix dialysis buffer

Reagent	Final concentration	Amount
1 M HEPES-NaOH (pH 7.4)	20 mM	20 mL
5 M NaCl	50 mM	10 mL
3 M KCl	10 mM	3.33 mL
1M MgCl_2_	1 mM	1 mL
100 mM TCEP	0.5 mM	5 mL
ddH_2_O	N/A	Add to 1000 mL
**Total**	**N/A**	**1000 mL**

Store at 4°C. This buffer should be freshly prepared.

## Step-by-step method details

### Preparation of SeEN-seq DNA library

**Timing: 5–6 h**

This section describes the preparation of a SeEN-seq DNA library by EcoRV digestion followed by gel purification.

1. Briefly spin down the 96-well plates containing dried DNA.
2. Add 10 μL of TE buffer to each well and resuspend the DNA.
3. Seal the plates and spin down.
4. Pool equal amounts of DNA from each well into a 1.5 ml tube.

***Note:*** Prepare a table to determine how much DNA to take from each well in advance.

5. Measure the DNA concentration with Nanodrop.

***Note:*** If pooling 700 ng (0.7 μg) from each 139 DNA construct, the theoretical quantity of DNA is: 0.7 (ug) x 139 (construct) = 97.3 μg

**Pause point:** Store DNA samples at −20°C until DNA digestion.

6. Digest 30 μg of the pooled DNA with EcoRV-HF (New England Biolabs). Store remaining DNA at −20°C for backup. The composition of EcoRV digestion is as follows:

**Table:** 

Reagent	Final concentration	Amount
Pooled DNA library	0.07–0.1 μg/ml	30 μg
10x Cutsmart buffer	1x	-
EcoRV-HF	-	Recommended usage according to the manufacturer’s instructions

***Note:*** See manufacturer’s instructions from the following link: https://www.neb.com/en-gb/protocols/2012/12/07/optimizing-restriction-endonuclease-reactions

7. Incubate samples in a 1.5 ml tube at 37°C for 1h.
8. Prepare a 3% agarose gel containing SYBR Safe (1:10,000 dilution).
9. After incubation, check DNA digestion by loading samples on the gel.
  a. 100 bp DNA ladder
  b. 10 μL of undigested DNA mixed with 2 μL of 6x loading dye
  c. 10 μL of digested DNA mixed with 2 μL of 6x loading dye
10. Once complete digestion is confirmed, load all samples into a 3% agarose gel containing SYBR Safe (1:10,000 dilution) to separate adaptor DNA (73 and 74 bp).

***Note:*** Use a large comb to load 40–60 μL in each well if needed to reduce the amount of gel.

**Figure 2:**
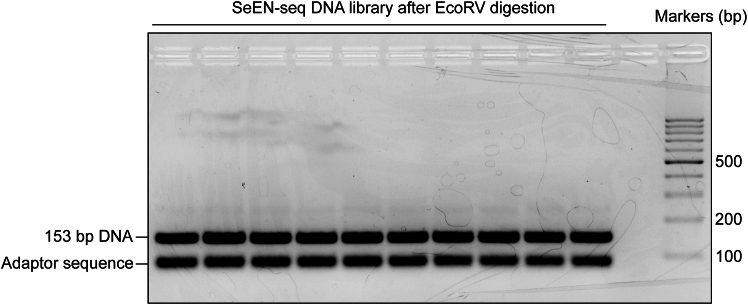
A representative gel image showing DNA fragments after EcoRV digestion
12. Purify each gel slice using QIAquick Gel Extraction kit (Qiagen) according to the manufacturer’s instructions (https://www.qiagen.com/gb/resources/resourcedetail?id=a72e2c07-7816-436f-b920-98a0ede5159a&lang=en).

***Note:*** Use one column per gel slice (e.g., 12 slices = 12 columns). To maximize DNA recovery, we used one column per gel slice to avoid exceeding the binding capacity of the column, which is limited by both the amount of gel solubilized and extracted DNA.

11. Excise the bands corresponding to 153 bp DNA (upper band) and purify with gel extraction (Figure 2).
  a. Place a gel on a plate wrapped in plastic wrap to prevent contamination.
  b. Excise the band corresponding to 153 bp DNA with a clean razor and transfer gel slices into new 1.5 mL tubes.

13. Pool all eluted DNA into a new 1.5 mL tube.
14. Further purify and concentrate the pooled DNA using QIAquick PCR Purification Kit (Qiagen) and elute in 30 μL of H_2_O.
15. Measure DNA concentration using absorbance at 260 nm.
16. Store purified DNA at −20°C.

***Note:*** In our previous result, we obtained 6.4 μg (42% yield as 153 bp). If the yield is insufficient, repeat the procedure with the remaining undigested DNA and combine all. Troubleshooting.

### Reconstitution of nucleosome library

**Timing: 3 days**

Core histones and reconstituted histone octamers are prepared according to the previously published protocols.^12^ For the nucleosome reconstitution, the SeEN-seq DNA library is spiked with an excess of carrier W601 DNA at around a 1:30 molar ratio (SeEN-seq DNA library: W601).

17. The composition of nucleosome reconstitution is as follows:

**Table:** 

Reagent	Volume (μL)	Final concentration
3 μg of SeEN-seq DNA library	X	0.4 mg/ml
97 μg of W601 carrier DNA	Y	
4M KCl	125	2 M
Histone octamers	Z	1: 1.8 (153 bp DNA: histone octamer)
ddH_2_O	125-X-Y-Z	
Total	250

***Note:*** Mix these compositions following order: water, 4 M KCl, DNA, and histone octamer. Do not chill 4 M KCl; Precipitation may occur.

**CRITICAL:** The DNA-to-histone ratio is crucial for successful nucleosome reconstitution. Too low and too high ratios result in increasing hexasome formation and precipitation, respectively. Highly recommend a small-scale test with W601 DNA to determine the best ratio. Perform a small-scale titration using W601 DNA to optimize conditions. The optimal DNA:histone octamer molar ratio is typically 1:1.6–2.0. Alternatively, nucleosomes can be reconstituted using H2A–H2B dimers and H3–H4 tetramers.^12^

18. Salt dialysis method for the nucleosome reconstitution follows a previous protocol.^12^
19. Transfer the reconstitution sample into a 1.5 ml tube.
20. Centrifuge at 5,000 rpm (2,348 x g) for 5 min at 4°C.
21. Transfer the supernatant into a new 1.5 ml tube.

***Note:*** A small pellet may be visible.

22. Measure DNA concentration by absorbance at 260 nm.

***Note:*** Typically, DNA concentration is around 0.2–0.3 mg/mL.

23. Check the quality of samples by loading samples on 6% non-denaturing PAGE (0.5xTBE):
  a. 100 bp DNA ladder.
  b. 150 ng nucleosomes + 5% sucrose.
24. Stain the gel with SYBR Safe (Invitrogen).
25. Keep reconstituted nucleosomes at 4°C.

### Purification of nucleosome library

**Timing: 5–6 h**

We purify the nucleosome library to remove free DNA and excess histone octamer. Here, we describe two different purification methods using either the anion exchange column or Prepcell apparatus.

***Note:*** For nucleosome purification with an anion exchange (Q) column, see Step 26. For DNA purification using the Model 491 Prepcell apparatus, see Step 27.

26. Nucleosome purification using an anion exchange column.
  a. Load sample onto an anion exchange column MonoQ 5/50 GL (Cytiva) using Buffer A: 20mM Tris-HCl pH 7.5.
  b. Elute the sample using a gradient from 30% to 80% Buffer B: 20 mM Tris-HCl pH 7.5, 1 M NaCl.
  c. The octameric nucleosome characteristically elutes ∼43 mS/cm, while the hexamer ∼50 mS/cm and the ‘free DNA’ at 57 mS/cm (Figure 3A).

**Figure 3:**
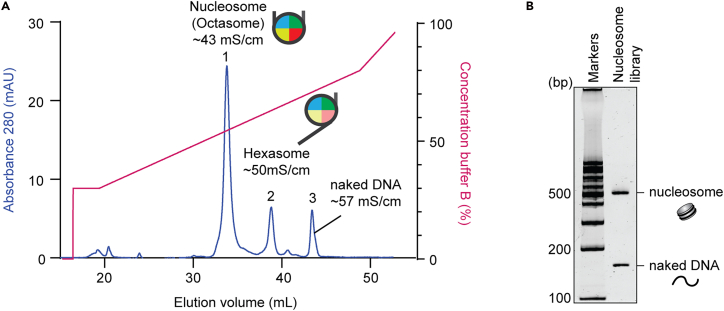
Ion-exchange chromatography separates octameric nucleosomes from the reconstitution mixture (A) Representative elution profile of a nucleosome purification following reconstitution using a 1mL MonoQ anion exchange column. Nucleosomes containing an octamer of histones (Octasome) elute at the lowest ionic strength (peak 1; ∼43 mS/cm conductivity). Hexasome, missing a single copy of H2A-H2B elute later (peak 2; ∼49mS/cm), with naked DNA of ∼150 base pairs, elutes at the highest ionic strength (∼53 mS/cm). (B) A representative gel image showing nucleosome library.
  d. Combine all fractions within the corresponding nucleosome peak and concentrate to ∼100 μL using an Amicon spin-filter (30K MWCO).
  e. Leave to dialyze into low salt buffer A ∼500 mL in the cold room overnight (>16 hours).
    **CRITICAL:** Do not dilute the nucleosome too much at this stage, as it may disassemble, especially in the high salt elution buffer.
  f. Collect the sample from a dialysis bag and concentrate it with an ultra-centrifugal filter (30 kDa cutoff).
  g. Measure DNA concentration by absorbance at 260 nm.
  h. Store on ice or 4°C.
27. Nucleosome purification using the Model 491 Prepcell apparatus.
  a. Prepare a 6% non-denaturing polyacrylamide gel in 0.2xTBE with a gel height of approximately 6.5 cm. Use TCS buffer EDTA (−) for sample elution.
    ***Note:*** Omit EDTA from elution buffer to prevent chelation of essential divalent cations such as Mg^2+^ and Zn^2+^.
  b. Pre-run the gel at 10 W for 1 h at a flow rate of 1.0 mL/min in the cold room.
  c. Load the nucleosome sample mixed with 5% sucrose into the Prepcell and start the run at 10 W (defined as time point 0 min).
  d. Collect the samples with 9 mL/fractions at a flow rate of 1.0 mL/min from 0 to 60 min.
    Collect the samples with 2.25 mL/fraction at a flow rate of 1.5 mL/min from 60 to 120 min.
    ***Note:*** Typically, nucleosomes will be eluted around 90 min.
  e. Analyze collected fractions by a 6% non-denaturing PAGE (0.5xTBE), running at 150 V for 1 h.
  f. Pool the fractions containing the nucleosome and concentrate them using an ultra-centrifugal filter (30k MWCO).
  g. Measure DNA concentration by absorbance at 260 nm.
  h. Store on ice or 4°C.
    ***Note:*** Recommended final concentration: > 0.1 mg/ml. Purified nucleosomes can be stored for 2–3 weeks on ice. To ensure the quality of samples immediate use is strongly recommended to ensure sample integrity. A representative gel image is shown in Figure 3B.

### SeEN-seq assay

**Timing: 2 days**

Using purified nucleosome libraries, perform an electron mobility shift assay (EMSA) to separate TF- bound and unbound nucleosomes. This is followed by gel extraction, DNA purification, and DNA library preparation for next-generation sequencing.

28. Prepare clean glass plates to cast 4.5%–6% non-denaturing PAGE in 0.5xTBE. The optimal gel percentage depends on the migration of your TF-nucleosome complex.

***Note:*** Lower percentage gels (4.5%–5%) tend to adhere to a glass plate. To reduce sticking, treat the glass plates with a cleaning concentrate (BIO-RAD) before gel casting.

29. Below is an example of a SeEN-seq assay. Final reaction buffer is 20 mM Tris-HCl (pH 7.5), 120 mM NaCl, 1 mM MgCl_2_, 10 μΜ ZnCl_2_, 1 mM DTT, and 100 μg/ml BSA. Zn^2+^ ions are especially important for zinc-finger proteins.

**Table:** 

	Condition 1 (no TF)	Condition 2 (0.8 μM ESRRB)	Condition 3 (1 μM ESRRB)
Reagents	Volume (μL)		

10x EMSA buffer BSA(+)	1	1	1
1 μM nucleosome library	1	1	1
Protein storage buffer	3	0.6	0
3.3 μM ESRRB	0	2.4	3
H_2_O	5	5	5
**Total**	10	10	10

***Note:*** Example of SeEN-seq assay using mouse orphan nuclear receptor Estrogen Related Receptor Beta (ESRRB).

***Note:*** Salt concentration (e.g. NaCl or KCl) is a critical factor for TF-nucleosome interactions. We recommend performing EMSA in 100-150 mM NaCl/KCl to approximate physiological conditions.

The DNA-binding domain may be sufficient for SeEN-seq. We recommend using full-length proteins whenever possible because the non-DNA-binding domain in some transcription factors may contribute to DNA binding specificity^13^^,^^14^ and interaction with histones.^3^

**CRITICAL:** To evaluate reproducibility of the sequencing data, perform three or more independent biological replicates at the same TF concentration. Testing different TF concentrations may help reveal distinct enrichment patterns.

30. Mix the reagents, nucleosomes, and transcription factors in a 1.5 mL DNA LoBind tube (Eppendorf) on ice.
31. Incubate samples at room temperature (20°C–25°C) for 30 min.
32. Add 2 μl of 30% sucrose to each sample.
33. Apply all samples to non-denaturing PAGE, running 100V for 1h. Running condition can vary based upon temperature, gel percentage and desired separation.

***Note:*** Leave one empty lane between samples to prevent cross-contamination.

**Figure 4:**
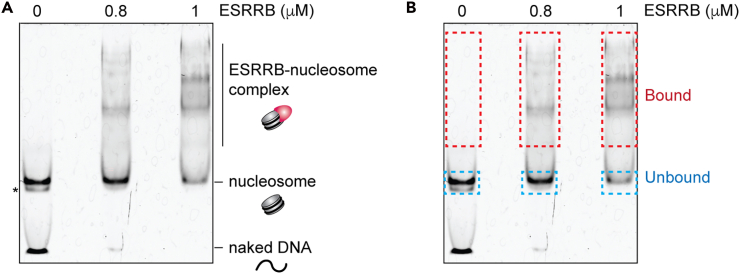
Gel excision to separate bound and unbound fractions (A) A representative gel image of EMSA. Leave one empty lane between samples. An asterisk indicates hexasome. (B) Dotted squares show the regions for gel excision.

***Note:*** You can include smear bands as bound fractions. In the negative control (no TF condition), excise the same gel area corresponding to the bound fraction as background. Include hexasome bands in the unbound fraction if visible. Troubleshooting.

34. Carefully open the glass plates and stain the gel in 100 mL of deionized water with 10 μL of SYBR Gold (Invitrogen) for 10 min.
35. De-stain with 100 mL of deionized water for 5–10 min.
36. Excise gel bands.
  a. Place a gel on a plate wrapped in plastic wrap to avoid contamination.
  b. Excise unbound and bound regions with a clean razor (Figure 4). Transfer a piece of gel into a new 1.5 mL DNA LoBind tube.

37. Spin down gel pieces to the bottom at 13,000 rpm (15,871 x g) for 1 min.
38. Crush gel with a small scalpel against the wall of the tube, resulting in small gel pieces.
39. Spin down again briefly to collect fragments.
40. Add 100 μL of gel extraction buffer.
41. Incubate at 50°C for 30 min on the heat block.
42. Add 450 μL of Qiagen Solubilization buffer and 50 μL of nuclease-free water (premixed).
43. Incubate at 50°C for 30 min on the heat block.
44. Spin down gel pieces at 13,000 rpm (15,871 x g) for 2 min.
45. Transfer the liquid to QIAquick spin columns (Qiagen).
46. Centrifuge at 13,000 rpm (15,871 x g) for 1 min.
47. Add 750 μL of PE buffer (Qiagen) and centrifuge at 13,000 rpm (15,871 x g) for 1 min.
48. Discard flow-through and centrifuge at 13,000 rpm for 2 min to remove residual PE buffer.
49. Elute DNA in 24 μL of nuclease-free water.
50. Measure DNA concentration by Qubit.

**Pause point:** Store DNA samples at −20°C until library preparation is performed.

51. Prepare DNA libraries with NEBNext Ultra II DNA Library Prep Kit for Illumina, according to the manufacturer’s instructions (https://www.neb.com/en-gb/-/media/nebus/files/manuals/manuale7103-e7645.pdf?rev=de09eaf8fcdf45e0ac8a66bf6fee75fb&hash=80FA51FEF16A47E60A9C0A09B1F745E2).

***Note:*** No more than 10 cycles of PCR amplification.

52. Measure DNA concentration using Qubit.
53. Check DNA fragment size using a Tapestation.
54. Sequencing on Illumina platform using 2 × 150 bp paired-end mode at a depth of 400K-800K reads per sample. Sample in this case means each sample extracted at the end of Steps 36–51.

### SeEN-seq data analysis preparation

**Timing: 40 min**

Here, we describe steps for the preparation of SeEN-seq data analysis.

**Figure 5:**
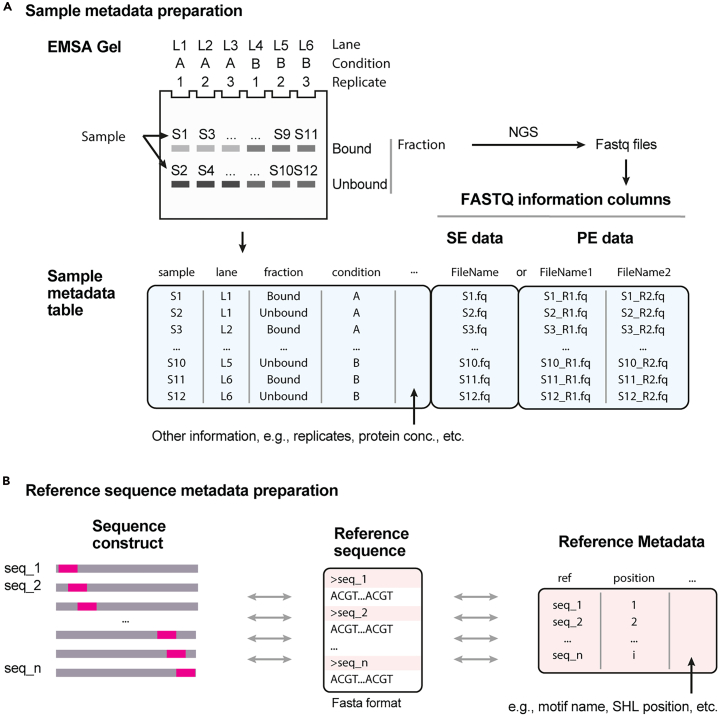
Schematic of sample (EMSA gel band) and reference sequence representation for the SeEN-seq analysis (A) Sample table representation of each fraction (band) on the EMSA gel. The FASTQ fields depends on whether the input data is single end (SE) or paired-end (PE) layout. (B) Sequencing reference metadata representation of the sequence construct used for nucleosome reconstitution.

***Note:*** The analysis of SeEN-seq datasets requires the following inputs: (i) raw reads sequence in FASTQ format, (ii) sample metadata table for each sample, (iii) reference sequences used for the experiment in FASTA format, and (iv) reference sequence metadata table. The FASTQ files are generated in the previous steps, and reference sequence can easily be converted to the FASTA format from the construct sequence. The guideline and constrains applied to the sample and reference sequence metadata tables is shown in Figure 5. For the purpose of reproducibility, all example FASTQ file inputs have been uploaded to Sequence Read Archieve (SRA) database. The non-sequencing input files have been deposited in the GitHub repository.

***Note:*** The list of tools required for this analysis is specified in the SeEN_seq_environment.yaml file of the repository. These tools can be manually installed, or installed using conda or similar environment managing tools. Note that the ‘x86_64’ distribution of conda is recommended, as some of the R packages may not be available in other distribution at the time of writing this protocol. We will start by environment set up as well as acquisition of the analysis scripts and example data.

55. Download the SeEN-seq analysis scripts from GitHub. Run the following command in terminal:

> git clone https://github.com/TotipotencyLab/SeEN_seq

> cd SeEN_seq

56. Set up environment (optional step). Troubleshooting.

> conda env create -f ./env/SeEN_seq_environment.yaml

> conda activate SeEN_seq

***Note:*** This step can be skipped if all the tools are available in your system. This environment also includes SRA-Toolkit to download the example dataset in the next step.

57. Download example dataset from Sequence Read Archive (SRA).

> x=$(cut -f 1 ./example/PRJNA1305216_SraAccList.txt)

> for acc in $x; do

>   echo $acc

>   fastq-dump --split-3 --gzip --skip-technical --outdir ./example/fastq/ $acc

> done

***Note:*** Preinstallation of SRA-Toolkit is required to use the fastq-dump command. A full list of SRA Run ID used as an example can be found in the example/PRJNA1305216_SraAccList.txt file. Alternatively, individual FASTQ file can be downloaded directly from the SRA website under the BioProject accession ID PRJNA1305216.

58. Prepare sample input metadata table.
  ***Note:*** This should be a file in tab-separated value (TSV) format. The example input file can be found in example/sample_metadata_PE.tsv of the GitHub repository. The sample metadata should consist of the following columns:
  a. 'sample': A unique name of your sample. This should correspond to each fraction isolated from the gel.
  b. 'lane': Information of which lane of the EMSA gel that the samples are from.
  c. 'fraction': Annotation of the fraction type of each sample. In this example, bound and unbound fractions.
    ***Note:*** In some cases, it is possible to have multiple fraction types within the same lane. For example, when multiple bound fractions are detected within the same lane of the gel.
  d. 'condition': Condition associated with the sample. This will be used to group the results from different groups together when the plot is made.
  e. Columns indicating the file paths to the FASTQ files associated to each sample. The standard column names used in QuasR package are used in this protocol. The names of the columns differ depending on the sequencing layout of the input:
    'FileName': For single-end input
    'FileName1' and 'Filename2': For paired-end input (this example)
  f. Other columns that may contain information related to the experimental variable of each sample, such as biological replicate number or input protein concentration (optional).
59. Prepare reference sequence metadata table (critical step).
  ***Note:*** This should be a file in tab-separated value (TSV) format. The example input file can be found in example/ref_metadata.tsv of the GitHub repository. It should consist of the following columns:
  a. ref: Name of the reference sequence.
  b. position: Position of the inserted transcription binding motif in the reference. This will be used as the X axis of the enrichment plot.
    **CRITICAL:** The ref column must have the same name as the sequence name of the FASTA file.

### Read alignment

**Timing: 30 min to 1 h**

The steps below describe read alignment. The analysis of SeEN-seq dataset can be performed entirely in an R script.

***Note:*** In this protocol, the R package QuasR will be used to align raw read data as well as counting the mappable reads to each reference construct sequence.^10^ The count matrix of mappable reads for each sample originated from each fraction (columns) to each reference sequence (row) is generated as an output. We recommend using multiple CPU cores to speed up the read alignment step. Optionally, this step can be skipped in case an alternative read mapping and counting tools is used.

60. Load required R packages.

> # In R console

> library("tools")

> library("tidyverse")

> library("parallel")

> library("QuasR")

> library("Biostrings")

> library("GenomicRanges")

> library("SummarizedExperiment")

> library("writexl")

61. Declare variable related to the current analysis project.

> # General analysis project setting

> wd <- getwd() # assume we are in the GitHub directory

> config <- list(

 project_name = "Test_SeEN_seq",

 output_dir = "./results/Test_SeEN_seq",

 sample_table_path = "./example/sample_metadata_PE.tsv",

 ref_table_path = "./example/ref_metadata.tsv",

 ref_fasta_path = "./example/ref_sequence.fasta",

 n_core=8,

 enrichment=list(c("Bound", "Unbound")), # test vs background

 log2_pseudo_count=1

)

> # Make sure output folder exist

> dir.create(config$output_dir, showWarnings=FALSE, recursive=TRUE)

> # Make sure the paths in config are absolute

> for(i in seq_along(config)){

 if(grepl("(_path$)|(_dir$)", names(config)[i])){

  config[[i]] <- file_path_as_absolute(config[[i]])

 }

}

> QuasR_dir <- paste0(config$output_dir, "/QuasR")

> plot_dir <- paste0(config$output_dir, "/plot")

> dir.create(QuasR_dir, showWarnings=FALSE)

> dir.create(paste0(QuasR_dir, "/cache"), showWarnings=FALSE)

> dir.create(plot_dir, showWarnings=FALSE)

***Note:*** Number of CPUs used for the raw reads mapping and read count by QuasR package can be specified in the 'n_core' field of the config variable.

62. Prepare input table for read alignment with QuasR.

> setwd(wd)

> sample_df <- read.table(config$sample_table_path, sep="\t", header=TRUE, stringsAsFactors=FALSE)

> # Extract relavant columns for QuasR

> QuasR_fq_col <- list(SE="FileName", PE=c("FileName1", "FileName2"))

> QuasR_fq_col_flag <- sapply(QuasR_fq_col, FUN=function(x){all(x %in% colnames(sample_df))})

> QuasR_fq_col_found <- QuasR_fq_col[QuasR_fq_col_flag]

> # Report wrong input column

> if(length(QuasR_fq_col_found) != 1){stop("Incorrect sample table column names")}

> # Extract input columns for QuasR and save

> QuasR_input_path <- paste0(config$output_dir, "/", config$project_name, "_QuasR_input.tsv")

> QuasR_df <- dplyr::select(sample_df, all_of(QuasR_fq_col_found[[1]]), SampleName=sample)

> # Get absolute path of FASTQ file

> for(fq_col in unlist(QuasR_fq_col)){

 if(fq_col %in% colnames(QuasR_df)){

  QuasR_df[[fq_col]] <- sapply(QuasR_df[[fq_col]],

                   tools::file_path_as_absolute)

 }

}

> write.table(QuasR_df, file=QuasR_input_path, sep="\t", row.names=FALSE, col.names=TRUE, quote=FALSE)

***Note:*** As mentioned earlier, the sample metadata table is required to contain the columns named 'FileName1' and 'FileName2' when the input FASTQ files are paired-end, and column named 'FileName' when the inputs are single-end. Mixing of inputs from both single-end and paired-end is not allowed in this example.

63. Read alignment and count using QuasR.

> # Setup CPU cluster for parallel run

> cl <- NULL # placeholder for non-parallel run

> avail_cores <- parallel::detectCores()

> if((avail_cores > 1) && (config$n_core > 1)){

  use_core <- min(config$n_core, avail_cores, na.rm=TRUE)

  cl <- parallel::makeCluster(use_core, type = "FORK")

}

> # Alignment with QuasR

> setwd(QuasR_dir)

> QuasR_proj <- QuasR::qAlign(

  sampleFile = QuasR_input_path,

  genome = config$ref_fasta_path,

  aligner = "Rbowtie",

  projectName = config$project_name,

  alignmentsDir = QuasR_dir,

  alignmentParameter = NULL,

  splicedAlignment = FALSE,

  cacheDir = paste0(QuasR_dir, "/cache"),

 clObj = cl)

> # Making reference for read count

> ref_fa <- Biostrings::readDNAStringSet(config$ref_fasta_path)

> ref_gr <- GRanges(seqnames = names(ref_fa), ranges = IRanges(start = 1, end = width(ref_fa)))

> names(ref_gr) <- as.character(seqnames(ref_gr))

> # Count read mapped to construct

> count_matrix <- QuasR::qCount(proj = QuasR_proj, query = ref_gr, clObj = cl)

> count_matrix <- count_matrix[,which(colnames(count_matrix)!="width")]

> if(!is.null(cl) && inherits(cl, "cluster")) parallel::stopCluster(cl)

***Note:*** This alignment step by the qAlign function takes the longest run time. However, this is only needed to be run once, and accidental re-running of the qAlign function will not trigger re-alignment of already exist alignment files because the QuasR package keeps track of the files generated by this function.

64. Saving read count matrix (optional).

> setwd(wd)

> count_matrix_path <- paste0(config$output_dir, "/", config$project_name, "_count_matrix.txt")

> write.table(count_matrix, file=count_matrix_path, sep="\t", row.names=TRUE, col.names=TRUE, quote=FALSE)

### SeEN-seq post-alignment processing

**Timing: 30 min**

**Figure 6:**
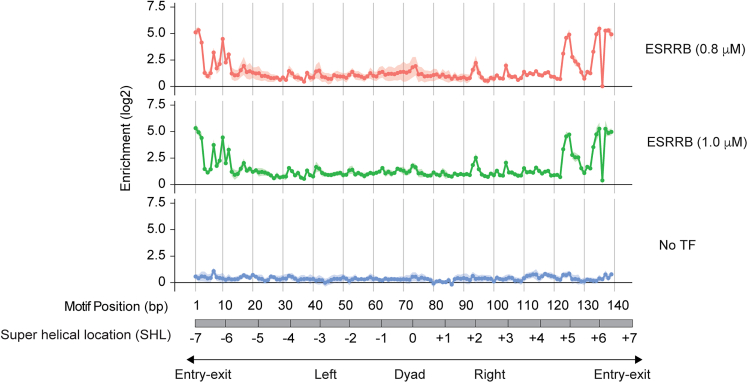
SeEN-seq profiles of ESRRB Enrichment values are plotted against the positions at which the Esrrb motif (TCAAGGTCA) starts along the W601 DNA and SHL. Data are shown as mean ± s.d. for three independent experiments.

The present section describes how to show the enrichment scores of transcription factor binding to each reference sequence are calculated as log2 value of the ratio of normalized counts between bound and unbound fractions (Figure 6).

***Note:*** The count matrix generated from the previous step, or independently generated from other alignment tools, can be used as an input for this step. As the analysis of SeEN-seq focuses on a comparison between the bound and unbound fraction of the same lane of the gel, the count matrix is first split according to the type of fraction from which it is derived. The columns of these matrices are renamed to the group of samples specified in the ‘lane’ column of the sample metadata. The count matrices and other matrices generated from this step will be stored in an R object class SummarizedExperiment to ensure that they have the same dimension.

65. Transform sample metadata to lane metadata (i.e., information for each lane in EMSA gel).

> # Count unique value of each column per 'lane'

> lane_col_unq_vals <- sapply(

  split(sample_df, f=sample_df$lane),

  FUN=function(df){

   sapply(df, function(x){length(unique(x))})

  })

> invalid_lane_df_colnames <- rownames(lane_col_unq_vals)[apply(lane_col_unq_vals > 1, MARGIN=1, FUN=any)]

> if(length(invalid_lane_df_colnames) > 0){message("These columns will be removed from the lane metadata table:\n ", paste0(invalid_lane_df_colnames, collapse=", "))}

> # Construct lane metadata table

> lane_df <- sample_df %>%

  dplyr::select(-all_of(invalid_lane_df_colnames)) %>%

  dplyr::distinct() %>%

  tibble::column_to_rownames(var="lane")

***Note:*** In this step, the sample table (fraction information) will be transformed to a lane table (lane information), which requires a non-redundant value in the lane column. To this end, some columns, in invalid_group_df_colnames variable, will be removed out of the original sample table. This should include fraction-specific columns, such as the 'sample' and the 'fraction' columns. Please make sure that important columns, such as 'condition', are not amongst the excluded columns.

66. Split count matrix by fraction types.

> fraction_names <- unique(sample_df$fraction)

> lane_name <- unique(sample_df$lane)

> # placeholder matrix for empty count

> zeros_mat <- matrix(0, nrow=nrow(count_matrix), ncol=length(lane_name), dimnames=list(rownames(count_matrix), lane_name))

> # Collecting reads

> count_mat_fraction <- list()

> for(i in seq_along(fraction_names)){

  cur_name <- paste0("counts_", fraction_names[i])

  sample_lane_map <- sample_df %>%

    dplyr::filter(fraction == fraction_names[i]) %>%

    dplyr::pull(lane, name=sample)

 m <- count_matrix[ , names(sample_lane_map), drop=FALSE]

  colnames(m) <- sample_lane_map[colnames(m)]

  missing_samples <- setdiff(lane_name, colnames(m))

 if(length(missing_samples) > 0){

  # Add missing samples with zero counts

  m <- cbind(m, zeros_mat[ , missing_samples, drop=FALSE])

 }

  m <- m + config$log2_pseudo_count

  # reorder the columns to match with lane_name

  count_mat_fraction[[cur_name]] <- m[ , lane_name, drop=FALSE]

}

***Note:*** A pseudo count is added here to prevent an infinite value originating from division by zero counts. This behavior can be changed in the config variable declared previously.

67. Store information to SummarizedExperiment object.

> ref_df <- read.table(config$ref_table_path, sep="\t", header=TRUE, stringsAsFactors=FALSE, row.names="ref_name")

> ref_df <- ref_df[rownames(count_matrix), ]

> SeEN_se <- SummarizedExperiment(assays=count_mat_fraction, colData=lane_df[lane_name , ], rowData=ref_df[rownames(count_matrix), ])

***Note:*** Other information related to each SeEN-seq experiment, such as config variable or unsplited read count matrix, can be added in the metadata field of the SummarizedExperiment object.

68. Library size normalization.

> for(i in seq_along(fraction_names)){

  m <- assay(SeEN_se, paste0("counts_", fraction_names[i]))

  lib_size_mat <- matrix(colSums(m), nrow=nrow(m), ncol=ncol(m), byrow=TRUE)

  cur_assay <- paste0("libnorm_counts_", fraction_names[i])

  assay(SeEN_se, cur_assay) <- m/lib_size_mat

}

69. Calculating enrichment score.

> for(i in seq_along(seq_along(config$enrichment))){

  frac_test <- config$enrichment[[i]][1]

  frac_ctrl <- config$enrichment[[i]][2]

  cur_assay<- paste0("enrichment_", frac_test, "_vs_", frac_ctrl)

  test_m <- assay(SeEN_se, paste0("libnorm_counts_", frac_test))

  ctrl_m <- assay(SeEN_se, paste0("libnorm_counts_", frac_ctrl))

  assay(SeEN_se, cur_assay) <- log2(test_m/ctrl_m)

}

70. Make temporary function for extracting data from SeEN_se object.

> SeEN_se_2_tidy <- function(se, assay_name, value_colname=NULL){

  if (is.null(value_colname)) value_colname=assay_name

  tidy_df <- as.data.frame(assay(se, assay_name)) %>%

   rownames_to_column(var="ref_name") %>%

   gather(key="lane", value=!!sym(value_colname), -ref_name) %>%

   # Adding metadata columns from lane and reference table

  left_join(rownames_to_column(as.data.frame(colData(se)), var="lane"), by="lane") %>%

  left_join(rownames_to_column(as.data.frame(rowData(se)), var="ref_name"), by="ref_name")

  return(tidy_df)

}

71. Checking whether all reference sequence are equally represented in the starting sample (unbound fraction of EMSA lane with no transcription factor added).

> # Extract raw counts from unbound fraction of lane with no TF added

> unbound_df <- SeEN_se_2_tidy(SeEN_se[, colData(SeEN_se)$Protein_conc_uM == 0], assay_name="counts_Unbound", value_colname="count")

> # Detecting outlier

> unbound_df <- unbound_df %>%

  dplyr::group_by(lane) %>%

  dplyr::mutate(

   outlier = count %in% boxplot.stats(count)$out,

   label = dplyr::if_else(outlier, true="Outlier", false="Normal"),

   outlier_label = dplyr::if_else(outlier, true=position, false=NA)

  ) %>% dplyr::ungroup()

>

> # Show under/over-represented construct for each lane

> print(dplyr::filter(unbound_df, outlier)[, c("lane", "position", "count")])

***Note:*** This step aims to identify reference constructs (identified by insert position of motif) that have abnormally low or high read count in their respective EMSA lane. Filtering of the SeEN_se object for lane with no protein added can be done in various way. For example, by column from sample metadata (Protein_conc_uM in this example), or by specifying exact target lane names. The boxplot.stats function is used to automatically detect reference construct with abnormal raw read count value. However, it is recommended to manually inspect validity of these suggested outlier constructs using the plot generated in the next step.

72. Visualizing reference sequence representation in starting sample.

> x_breaks <- c(1, seq(0, max(unbound_df$position, na.rm = T) + 10, by = 10)[-1])

> p_unbound <- unbound_df %>%

  ggplot(aes(x = position, y = count, group = position)) +

  geom_hline(yintercept = 0 + config$log2_pseudo_count) +

  geom_col(aes(fill = label), alpha = 0.5) +

  geom_point(aes(color = label)) +

  geom_text(aes(label = outlier_label), vjust = -1) +

  scale_y_log10() + scale_x_continuous(breaks=x_breaks) +

  facet_grid(lane ∼ ., scales = "free_y") +

  labs(title = "Raw reads from unbound fraction without TF") +

  theme_bw()

> ggsave(plot = p_unbound, filename = paste0(plot_dir, "/", config$project_name, "_unbound_count_plot.pdf"), width = 8, height = 5, dpi = 300, units = "in")

**Figure 7:**
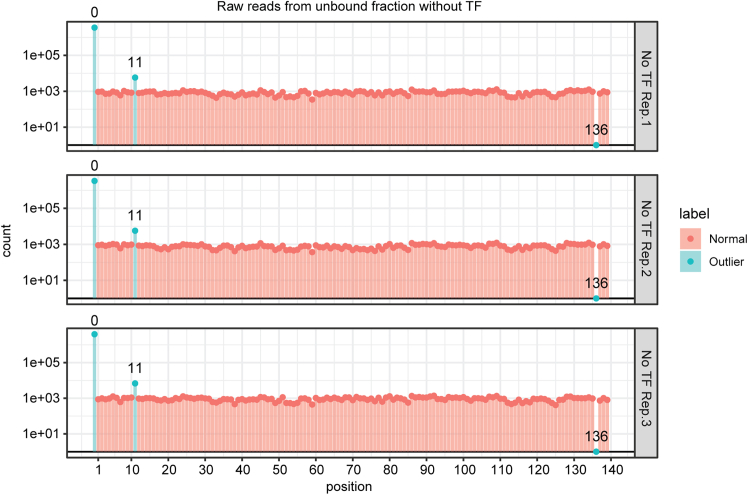
Raw reads number of the SeEN-seq nucleosome library Bar plot for detecting over- and underrepresented raw read counts of each reference sequence within the starting material (unbound fraction of lane with no TFs). Individual reference sequence is indicated by the start position of inserted ESRRB motif on the template (x-axis), where position zero corresponds to the template sequence with no motif. Row: three individual biological replicates.

***Note:*** The raw read counts representation is shown in Figure 7. The position 0 (carrier DNA without TF motif) was detected as an overrepresented construct, which is expected because carrier DNA contains a higher concentration for nucleosome reconstitution. Insertion of the ESRRB motif at the 136 bp position resulted in a sequence matching restriction site of EcoRV. Therefore, this sequence was omitted from the DNA library. The higher read number position 11 is likely due to human errors during library pooling. Troubleshooting

73. Calculate average enrichment across biological replicates.

> # Extract enrichment matrix and transform to tidy format

> tidy_enrich_df <- SeEN_se_2_tidy(SeEN_se, assay_name = "enrichment_Bound_vs_Unbound", value_colname = "enrichment")

>

> # Calculate average enrichment score across replicates

> enrich_df <- tidy_enrich_df %>%

  group_by(position, condition) %>%

  dplyr::reframe(

   avg_enrichment = mean(enrichment),

   sd_enrichment = sd(enrichment),

   sd_enrichment = replace(sd_enrichment, is.na(sd_enrichment), 0)

  )

74. Making plots.

> p <- enrich_df %>%

  dplyr::filter(position > 0) %>% # ignore the construct with no motif

  ggplot(aes(x=position, y=avg_enrichment, group=condition)) +

  geom_hline(yintercept=0, color="black") +

  geom_ribbon(aes(ymin=avg_enrichment-sd_enrichment,

     ymax=avg_enrichment+sd_enrichment,

     fill=condition), alpha=0.3) +

  geom_line(aes(color=condition)) +

  geom_point(aes(color=condition)) +

  scale_x_continuous(breaks=x_breaks) +

  scale_y_continuous(limits=c(NA, 7.5), breaks=seq(-0, 7.5, by=2.5)) +

  facet_grid(condition∼.) +

  labs(x="Position", y="Enrichment Score") +

  theme_classic() +

  theme(legend.position="bottom",

strip.text.y=element_text(size=12, angle=0),

   strip.background.y=element_blank())

   ggsave(plot=p, filename=paste0(plot_dir, "/", config$project_name, "_enrichment_plot.pdf"), width=8, height=5, dpi=300, units="in")

75. Save output data as files.

> # RDS object for future reanalysis

> saveRDS(SeEN_se, file=paste0(config$output_dir, "/", config$project_name, "_SE.rds"))

> # Save to Excel file

> result_list <- c(

  list(raw_count = tibble::rownames_to_column(as.data.frame(count_matrix), var="sample")),

  lapply(assays(SeEN_se), FUN=function(m){tibble::rownames_to_column(as.data.frame(m), var="sample")}),

  list(

   ref_metadata = rownames_to_column(as.data.frame(rowData(SeEN_se)), var="ref_name"),

   sampl_lane_metadata = rownames_to_column(as.data.frame(colData(SeEN_se)), var="ref_name"),

   sample_metadata = sample_df

))

> writexl::write_xlsx(result_list, path=paste0(config$output_dir, "/", config$project_name, "_results.xlsx"))

### Preparation of TF-nucleosome complex by Grafix

**Timing: 2–3 days**

To stabilize and purify the TF-nucleosome complex, we use a Grafix method.^15^ Here, we describe two different methods for Grafix and desalting.

76. Reconstitution of TF-nucleosome complex.
  a. Based on the SeEN-seq result, select several enriched positions. In the case of ESRRB (Figure 6), the motif positions around the super helical locations (SHLs) −6, +5, and +6 are preferentially targeted by ESRRB. For example, motif positions 10, 125, and 135 can be selected for reconstitution of the ESRRB-nucleosome complex.
  b. The selected DNA construct can be directly amplified from the SeEN-seq library plate and subcloned into a vector, such as pGEM-T easy (Promega). The selected DNA can then be amplified by PCR and purified as described above.
  c. Reconstitute and purify the W601 nucleosome containing TF motif. The composition of nucleosome reconstitution is as follows:

**Table:** 

Reagent	Volume (μL)	Final concentration
300 μg of W601 containing TF motif	X	0.8 mg/ml
4M KCl	187.5	2 M
Histone octamers	Y	1: 1.8 (153 bp DNA: histone octamer)
ddH_2_O	187.5-X-Y	
Total	375
  d. Prepare the TF-nucleosome complex. Example of the reconstitution of NR5A2-nucleosome complex as follows:

**Table:** 

Reagent	Final concentration	Volume (μL)
10x EMSA buffer BSA (−)	1x	40
8 μM W601 nucleosome containing TF motif	2 μM	100
25 μM human NR5A2	4 μM	64
Protein storge buffer	-	56
H_2_O	-	140
Total	-	400
    ***Note:*** Omit BSA from 10x EMSA buffer. To avoid an excessive amount of TF, choose the minimum ratio between nucleosomes and TF by EMSA. This ratio depends on the concentration. If your TF of interest shows aggregation, prepare complexes at low concentration and concentrate before sample loading.
  e. Incubate samples at room temperature (20°C–25°C) for ∼30 min. This can also be adjusted depending on the stability and affinity of your complex.
77. Purification of the TF-nucleosome complex by Grafix method.
  a. Create a gradient using low sucrose buffer and high sucrose buffer with a linear gradient master (BioComp). Here is the example for buffer composition for Grafix. The percentage of sucrose in the high and low sucrose buffer can be optimized for the size of the nucleosome-TF complex. See similar protocol in published papers.^4^^,^^16^^,^^17^
    ***Note:*** Can use either a SW40/41Ti swinging rotor for larger volumes (14 mL) or SW60 rotor for smaller volumes (4 mL)
    **Table:** 5% sucrose buffer

Reagent	Final concentration	Amount
1 M HEPES-NaOH (pH 7.5)	10 mM	0.5 mL
5 M NaCl	20 mM	0.2 mL
1 mM ZnCl_2_	1 μM	0.05 mL
1 M DTT	1 mM	0.05 mL
Sucrose	5%	2.5 g
ddH_2_O	N/A	Add to 50 mL
**Total**	**N/A**	**50 mL**

Store at room temperature (20°C–25°C). This buffer should be freshly prepared.

**Table:** 20% sucrose buffer with formaldehyde

Reagent	Final concentration	Amount
1 M HEPES-NaOH (pH 7.5)	10 mM	0.5 mL
5 M NaCl	20 mM	0.2 mL
1 mM ZnCl_2_	1 μM	0.05 mL
1 M DTT	1 mM	0.05 mL
Sucrose	20%	10 g
32% Formaldehyde	2%	3.125 mL
ddH_2_O	N/A	Add to 50 mL
**Total**	**N/A**	**50 mL**

Store at room temperature (20°C–25°C). This buffer should be freshly prepared.

**Table:** 20% sucrose buffer with glutaraldehyde

Reagent	Final concentration	Amount
1 M HEPES-NaOH (pH 7.5)	10 mM	0.5 mL
5 M NaCl	20 mM	0.2 mL
1 mM ZnCl_2_	1 μM	0.05 mL
1 M DTT	1 mM	0.05 mL
Sucrose	20%	10 g
25% glutaraldehyde	0.33%	660 μL
ddH_2_O	N/A	Add to 50 mL
**Total**	**N/A**	**50 mL**

Store at room temperature (20°C–25°C). This buffer should be freshly prepared.
    ***Note:*** Buffer components should not contain any primary amines, such as Tris, as they will react and quench crosslinking reagents.
  b. Load 200 μL of complexes into the top of each tube if using SW40, 14 mL tubes (or <100 μL if using SW60).
    ***Note:*** Keep a few μL at 4°C as input sample.
  c. Ultracentrifugation at 27,000 rpm (124,668 x g) for 16 h at 4°C using a Beckman SW-41Ti rotor. No brake for deceleration.
  d. Either fractionate with the BioComp fractionator (see manufacturer’s details: https://biocompinstruments.com/documents/type/category/manuals) or manually by carefully pipetting every 500 μL from the top. Store the samples at 4°C.
  e. Quench samples with 1 M Tris-HCl (pH 7.5, 100 mM final concentration) or glycine.
  f. Analyze the samples with 4.5%–6% PAGE (0.5xTBE), 150V for 1 h.
    ***Note:*** Formaldehyde affects sample migration.
  g. Stain the gel with SYBR Gold and image acquisition. Troubleshooting.
  h. Pool fractions containing TF-nucleosome complex.
78. Desalting.
  ***Note:*** Desalting the samples by either PD-10 column or dialysis; both methods work efficiently. The PD-10 column allows rapid buffer exchange and enables cryo-EM specimen preparation on the same day. For Cryo-EM specimen preparation, buffer composition may need to be optimized depending on your sample. The described buffer compositions were empirically determined in our previous works.
  a. Desalting the samples with PD-10 column.
    i. Prepare the samples with PD-10 elution buffer and equilibrate according to the manufacturer’s instructions (https://www.cytivalifesciences.com/en/us/products/items/sephadex-g-25-in-pd-10-desalting-columns-p-05778?selectedProduct=17085101).
    ii. Add 2.5 mL samples into each column.
    iii. Elute samples with 3.5 mL PD-10 elution buffer using gravity protocol.
    iv. Pool all eluted samples and concentrate with an ultra-centrifugal filter (30k MWCO).
  b. Desalting the samples by dialysis.
    i. Prepare the Grafix dialysis buffer and keep it at 4°C.
    ii. Dialyze the sample against Grafix dialysis buffer overnight (>16 hours).
    iii. Collect the samples and concentrate with an ultra-centrifugal filter (30k MWCO).
  c. Measurement of DNA concentration (absorbance 260 nm).
    ***Note:*** Recommend to concentrate 200–500 μg/ml (2–5 μM).

### Cryo-EM specimen preparation

**Timing: 2–3 h**

Vitrification of TF-nucleosome complexes for cryo-EM analysis.

79. Using a plunge-freezer (e.g., Vitrobot, Leica GP2, etc.) and follow the manufacturer’s instructions for standard cryo-EM sample preparation (https://documents.thermofisher.com/TFS-Assets/MSD/manuals/vitrobot-mk-iv-user-manual-pn103261.pdf).
  a. Set the plunge freezer environment at 4°C, 100% humidity.
  b. Glow discharge or plasma clean grids. In general, the most optimal grids for nucleosome-TF complexes are Quantifoil QF 1.2/1.3 Cu 300 or UltraAuFoil.
  c. Apply 3–4 μL of sample to grid and plunge freeze into liquid ethane.
  d. Prepare a number of 4–8 grids with different blotting conditions and sample concentrations to identify optimal freezing conditions for ice thickness and particle distribution.
80. Clip grids for loading into the Autoloader in TEM and downstream screening and data collection.

## Expected outcomes

The rotational positioning of DNA motifs influences TF access to DNA and histones within the nucleosome. Most TFs preferentially bind to regions near the entry and exit sites of nucleosomal DNA, where the DNA exhibits highly dynamics and accessibility. TFs can periodically access motifs located on solvent-exposed regions of the nucleosome. This protocol, SeEN-seq, maps all potential TF-binding sites on nucleosomal DNA and reveals the intrinsic binding preferences of TFs in the context of chromatin. A successful outcome is presented in Figure 6. The negative control (no TF) shows no specific enrichment (Figure 6, lower panel), whereas the presence of TF results in clear site-specific enrichment (Figure 6, upper panel). SeEN-seq can be broadly applied to biochemical and structural studies investigating TF engagement with nucleosomes.

## Limitations

To design a SeEN-seq DNA library, the TF motif must be known and well characterized by *in vitro* assay or ChIP-seq data. While SeEN-seq can be extended to analyze composite motifs, such as OCT4–SOX2, it requires careful optimization in motif design and spacing to accommodate cooperative or sequential TF binding. Since SeEN-seq is based on the W601 positioning sequence tiled with canonical TF motifs, it may not fully capture unique features of TF binding or DNA repositioning observed with native nucleosomes.^18^

## Troubleshooting

### Problem 1

Low yield of SeEN-seq DNA library.

### Potential solution

Ensure complete EcoRV digestion before gel purification by checking on an analytical gel. During gel extraction, add 6 volumes of Buffer QG (Qiagen) to the excised gel slices, following the manufacturer’s instructions precisely (https://www.neb.com/en-gb/protocols/2012/12/07/optimizing-restriction-endonuclease-reactions). Preparation of the SeEN-seq library by PCR is also feasible.^6^ However, it should be noted that certain DNA sequences may be susceptible to PCR bias, which can disrupt the equal representation of each DNA construct.

### Problem 2

No visible TF-bound fraction in EMSA.

### Potential solution

Perform a titration series with increasing concentrations of the transcription factor to determine optimal binding. Consider verifying TF purity, activity, and storage conditions.

### Problem 3

Failure to install conda environment for SeEN-seq analysis, some packages do not exist.

### Potential solution

Some of the conda packages listed in./env/SeEN_seq_environment.yaml file may not be available for certain conda distribution. For example, at the time of writing this, the R package, QuasR (bioconductor-quasr), is not available for conda distribution for osx-arm64 (Apple Silicon) platform. If applicable, a possible solution is to reinstall a conda distribution for alternative platform that is suitable for your operation system. Alternatively, missing packages have to be manually installed. For example, the missing R packages can be manually installed using the install.packages() or BiocManager::install() function.

### Problem 4

Missing DNA constructs in SeEN-seq results.

### Potential solution

Verify whether the TF motif replacement introduces an EcoRV site, which may not be incorporated nucleosome library. Also consider human or pipetting error during pooling. Check pooling table accuracy.

### Problem 5

No visible TF-nucleosome complex after Grafix.

### Potential solution

Ensure the gradient buffer does not contain any primary amines, such as Tris. Higher concentrations of formaldehyde may destabilize the TF-nucleosome complex by masking lysine residues essential for DNA binding. Consider testing alternative crosslinking reagents (e.g., DSS or BS3) or performing short-term fixation in a tube on ice.

## Resource availability

### Lead contact

Further information and requests for resources and reagents should be directed to the lead contact, Kikuë Tachibana (tachibana@biochem.mpg.de).

### Technical contact

Technical questions on executing this protocol should be directed to and will be answered by the technical contacts, Wataru Kobayashi (wkobayashi001@dundee.ac.uk) and Alicia K. Michael (alicia.michael@ista.ac.at).

### Materials availability

This study did not generate new unique reagents.

### Data and code availability

Raw SeEN-seq data of ESRRB nucleosome binding have been deposited on the Sequence Read Achieve database under the accession PRJNA1305216. Example analysis scripts and input files for SeEN-seq analysis can be found at https://doi.org/10.5281/zenodo.17665082.

## Acknowledgments

We thank R.H. Kim, A. Casper, and R. Gautsch for sequencing at the NGS facility (RRID:SCR_025746). K.T. is an Honorary Professor at the Department of Biology, Ludwig-Maximilians-University, Munich, Germany.

This study was funded by European Research Council grant ERC-CoG-818556 TotipotentZygotChrom (K.T.), Max Planck Society (K.T.), and ERC Starting Grant “ChromaChrono” 101162145 (A.K.M.).

## Author contributions

M.K. performed the experiments. W.K., A.K.M., S.R., and K.T. conceived the project and wrote the manuscript. All authors discussed the results and commented on the manuscript.

## Declaration of interests

The authors declare no competing interests.

## Supplemental information

Table S1. Example of the SeEN-seq nucleosome library, related to Step 1
